# Supplementary figures and images for: The kinase Isr1 negatively regulates hexosamine biosynthesis in S. cerevisiae
Source: PLoS Genet. 2020 Jun 24;16(6):e1008840. doi: 10.1371/journal.pgen.1008840 (PMC7340321; doi:10.1371/journal.pgen.1008840)

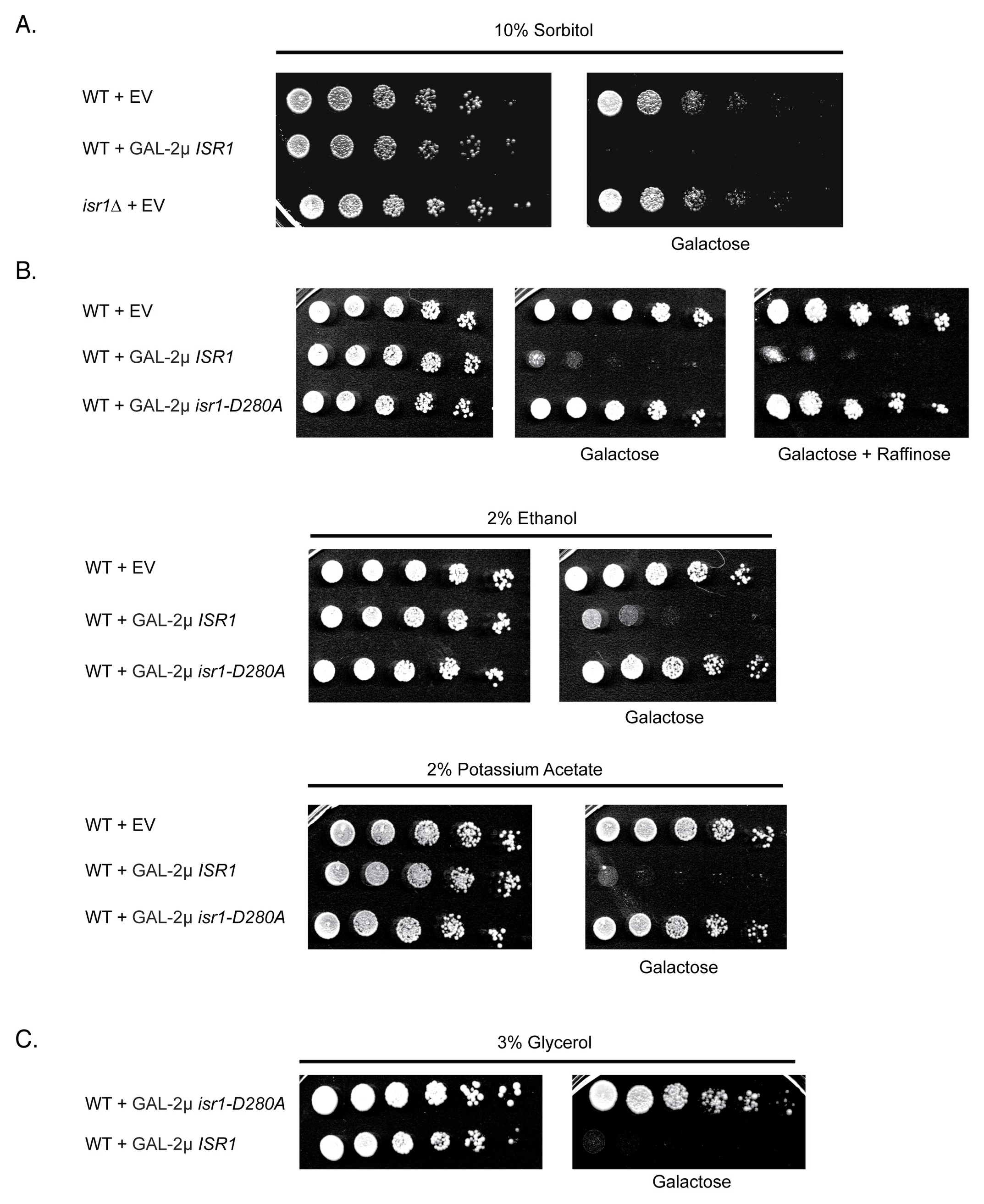

Supplement: S1 Fig — (A) Lethality of GAL-2μ ISR1 is not rescued by sorbitol. Cells of the indicated genotypes expressing EV or GAL-2μ ISR1 were diluted onto YPD or YPGal + 10% sorbitol. (B) Lethality from GAL-2μ ISR1 overexpression is not rescued by alternative carbon sources. Wild-type cells were transformed with EV, GAL- 2μ ISR1 or GAL- 2μ isr1-D280A and spotted onto CSM containing 2% glucose, galactose or the indicated carbon source. (C) Experiment performed as in B, but with 3% glycerol. (TIF) [file pgen.1008840.s001.tif]

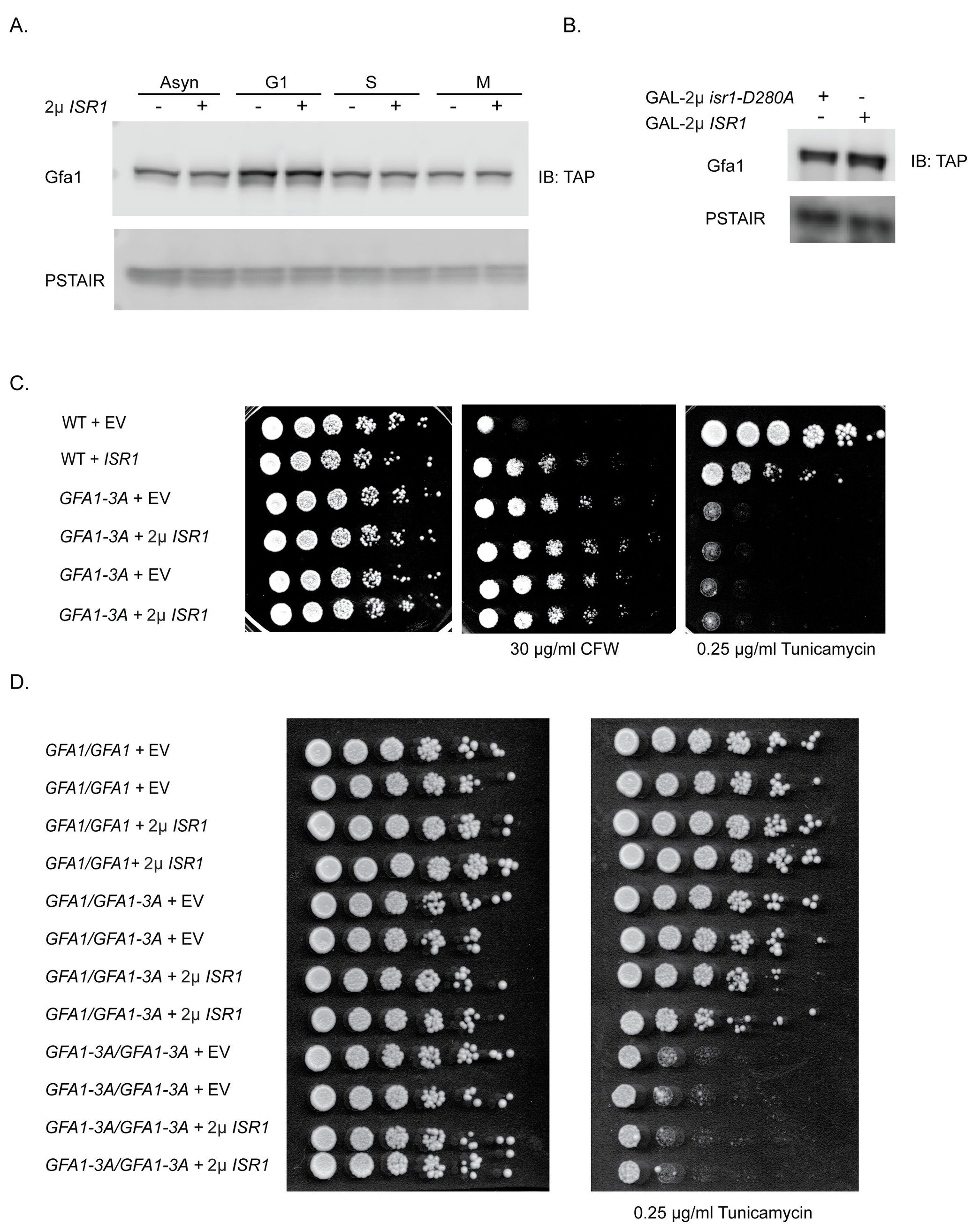

Supplement: S2 Fig — (A). 2μ ISR1 does not affect Gfa1 protein levels at any cell cycle stage. Cells were inoculated in CSM-URA and arrested in G1, S, and M phase with alpha factor, HU or nocodazole respectively. Note that Gfa1 was upregulated in response to alpha factor, as expected. (B) GAL-2μ ISR1 does not alter Gfa1 protein levels. Cells were grown overnight in CSM-URA raffinose and inoculated in CSM-URA galactose for 4 hours. (C) Cells of the indicated genotypes were transformed with EV or 2μ ISR1 and serial diluted on YPD with or without 30 μg/ml calcofluor white. (D) Diploids of the indicated genotypes were transformed with EV or 2μ ISR1 and spotted on YPD with or without 0.25 μg/ml tunicamycin. (TIF) [file pgen.1008840.s002.tif]

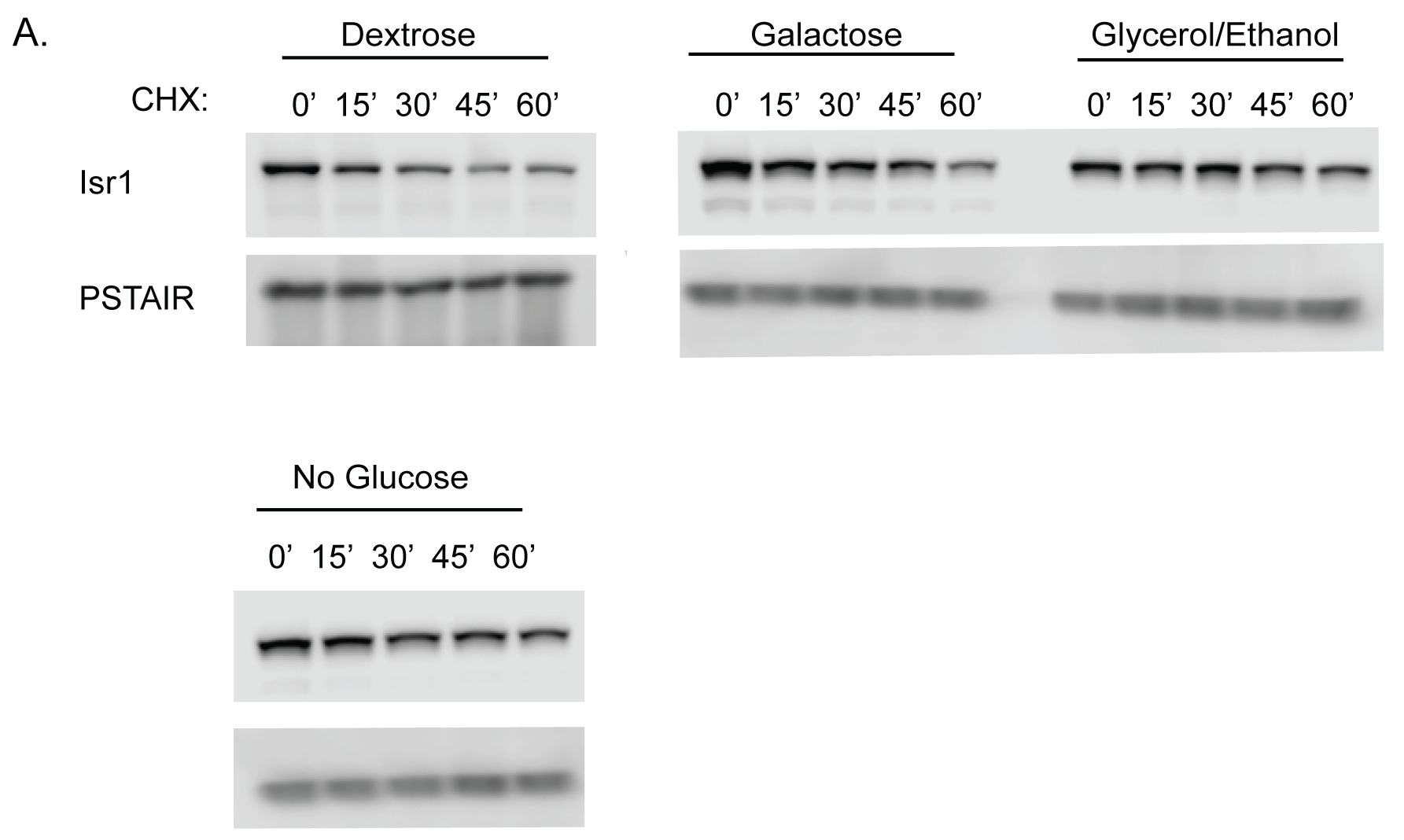

Supplement: S3 Fig — (A) Isr1 is partially stabilized by glycerol/ethanol, but not galactose. Cycloheximide-chase assay of Isr1-13xmyc grown in YM-1 containing 2% dextrose, 2% galactose or 2% glycerol/1% ethanol. Cells were grown overnight in the indicated carbon source, inoculated in fresh media, and cycloheximide was added for the indicated number of minutes. For the no glucose condition, cells were grown in YM-1 with dextrose, washed twice in media without a carbon source, and suspended in media with no carbon source at the same time as adding cycloheximide. (TIF) [file pgen.1008840.s003.tif]

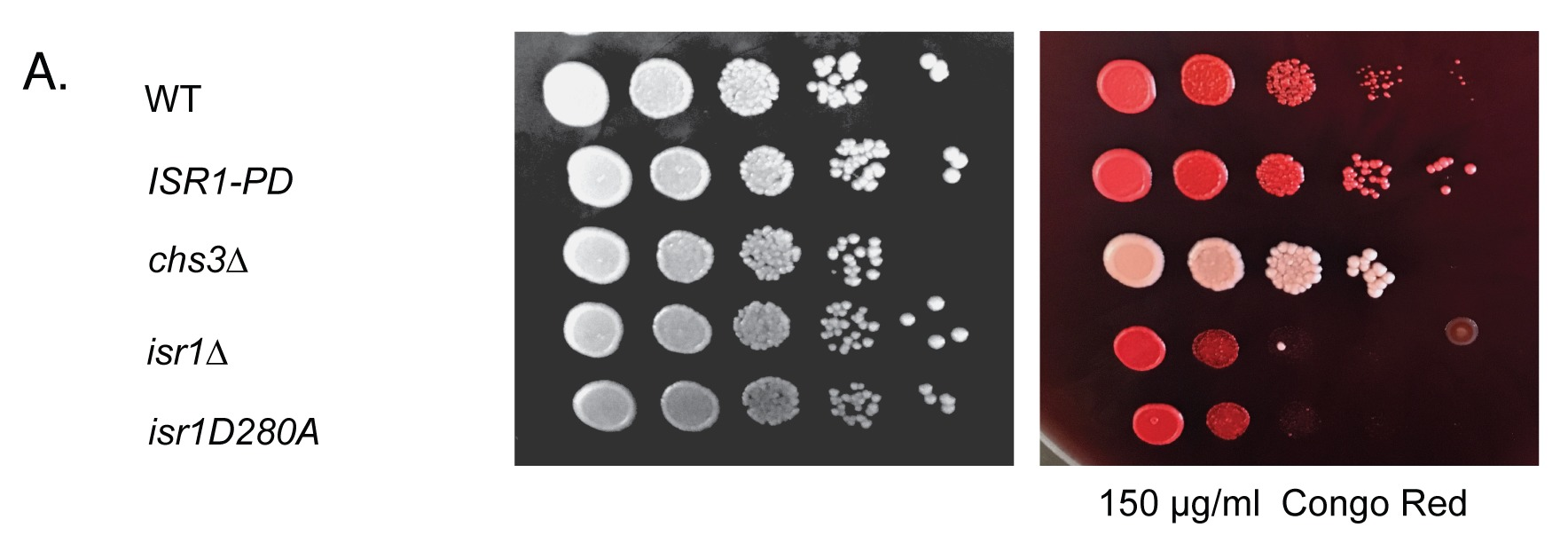

Supplement: S4 Fig — (A) An Isr1 phosphodegron mutant is resistant to Congo Red. Strains of the indicated genotypes were diluted onto YPD in the presence or absence of 150 μg/ml Congo Red. (TIF) [file pgen.1008840.s004.tif]

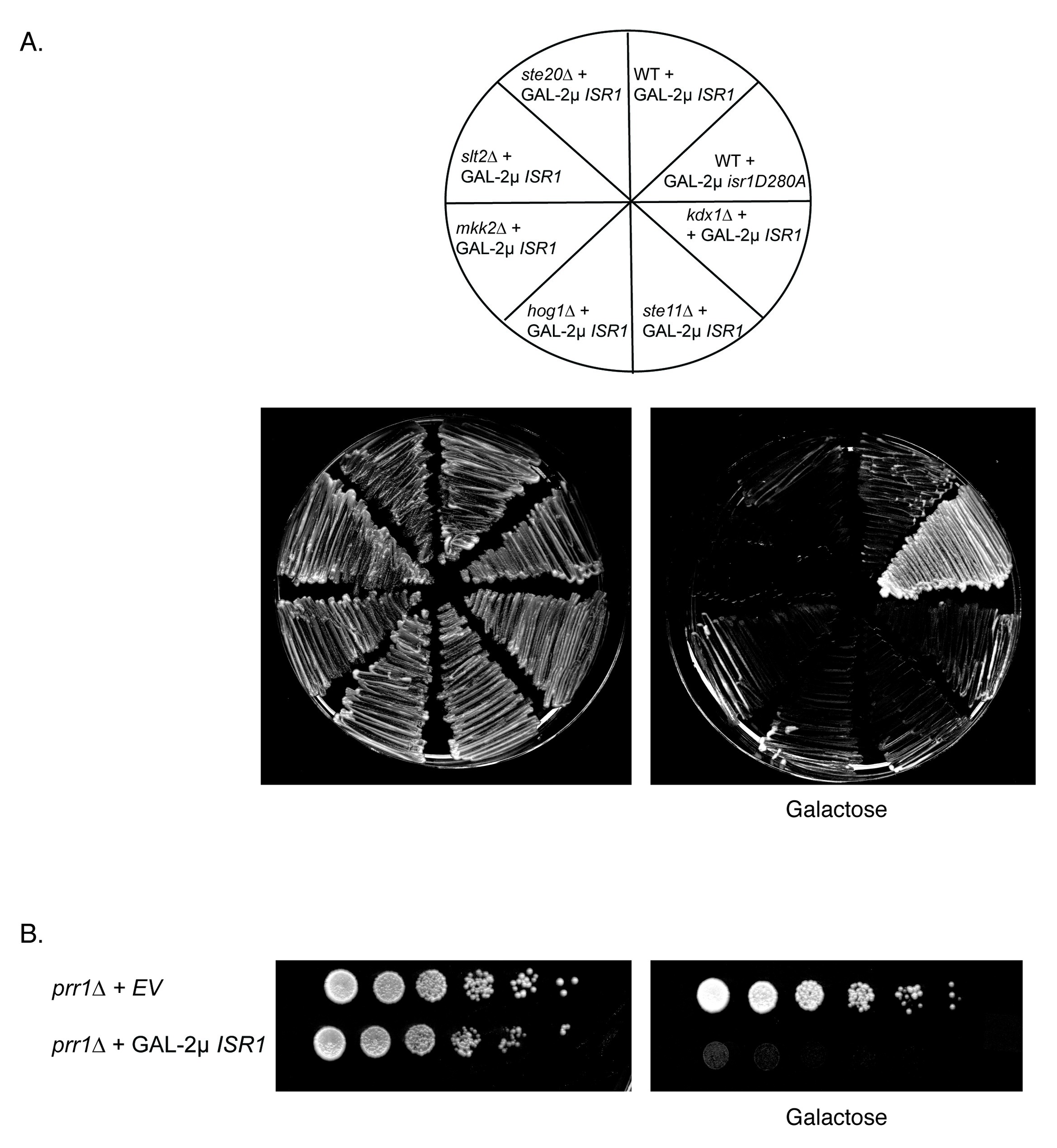

Supplement: S5 Fig — (A) Strains of the indicated genotypes expressing Gal- 2μ ISR1 or GAL-2μ isr1-D280A (wild-type only) were struck on YPD or YPGal plates. (B) ppr1Δ cells expressing EV or GALl-2μ ISR1 were serial diluted onto YPD or YPGal. (TIF) [file pgen.1008840.s005.tif]
