# Supplementary material for: The kinase Isr1 negatively regulates hexosamine biosynthesis in S. cerevisiae
Source: PLoS Genet. 2020 Jun 24;16(6):e1008840. doi: 10.1371/journal.pgen.1008840 (PMC7340321; doi:10.1371/journal.pgen.1008840)
Supplement: S6 Fig — Double-stranded DNA sequence used to generate ISR1 phosphodegron mutant. Blue sequence is the end of the ISR1 promoter. Lowercase red base pairs indicate mutated residues. (DOCX) [file pgen.1008840.s006.docx]

**S6 Fig. *ISR1-PD* sequence**

GTGATGTATGGGTCTATCTTATTGTTTATTTTGACTAGCATTGAATAAACAAAAAGCGCTGCTAATAGATTCTTGCCTTTATATAACGGGGATCTAACCTCATCAACAGCAAAAATCGTCTTAAAACACATCTCAAAGACTAGTTCTCAAACGTCACGCTATGAACgctgCACCTCCTgccgCACCCGTCACCAGGGTTTCTGATGGTTCCTTTCCATCCATAAGTAACAATAGTAAGGGTTTTGCTTATCGCCAACCGCAAAAACATAAAAGTAACTTCGCATATTCACATCTGGTATCTCCTGTAGAGGAGCCGACAGCTAAATTCAGTGAGGCATTCCAGACAGATTATTCTAGTAAGGCGCCCGTTGCTACCTCGGAGGCGCACCTAAAGAACGATTTAGACGTATTGTTCgCTgCCCCCCGGTTTTACgCTCCGGAGAATTTGGCTTTAATGTTCCGTCTTTCTAATACAGTTTCTTCCCTAGAATTTCTGGATGAGTTTTTGATGGGCATATTACTTGCTCCAGAGATGGATTTTTTGTCAAATCCAAGTTATTCTCTTCCGTCTAACAAATTAGTGGGACAGGGAAGTTATTCATATGTGTACCCTATATCATCAAGTGCTTCATCAAGATGTAACAACGATTCAGGGGTTGTTTTAAAGTTTGCCAAATCACAGCATAAAAGCAAGGTGATTTTACAGGAAGCTTTGACGCTAGCATATCTCCAGTACATGAGTCCTTCAAC
