# Supplementary material for: The kinase Isr1 negatively regulates hexosamine biosynthesis in S. cerevisiae
Source: PLoS Genet. 2020 Jun 24;16(6):e1008840. doi: 10.1371/journal.pgen.1008840 (PMC7340321; doi:10.1371/journal.pgen.1008840)
Supplement: Attachment — Submitted filename: Plos Genetics response to reviewers .pdf [file pgen.1008840.s010.pdf]

Dear Dr. MacGurn,

We thank the reviewers for the careful reading of our paper, and we were happy that they felt that the work is significant and impactful. We have now altered the manuscript in several ways, detailed below, in response to the reviewers' comments. Specifically, we have: 1. Removed references about homology to RAF. 2. We have now quantified the western blots in Figures 1 & 5 and present these quantifications below the western blots. 3. We clarify the rational for the use of the PSTAIR antibody. We hope that you will find these changes acceptable.

Sincerely,

Emma Alme and David Toczyski

Reviewer #1.
Summary. Yeast encode 117 protein kinases in the superfamily of eukaryotic protein kinases, plus a smattering of atypical protein kinases related in function but not evolutionary lineage. Almost all of the yeast kinases have been characterized at least to some extent. This paper determines the function of one of the few remaining poorly characterized protein kinases in yeast. The experiments presented here show the following 1) they demonstrate the role of ISR1 in regulating the hexosamine pathway; 2) they use phosphoproteomic data to identify GFA1 as the ISR1 substrate and 3) they show the regulation of ISR1 abundance via a PHO85-targeted phosphodegron. These results are significant and novel. A few changes to the manuscript that could strengthen the paper are noted below.

1. ISR1 and Raf/Ras signaling. The first paper to describe ISR1 is the Miyahara paper from 1998. This paper states that ISR1 "has the highest degree of similarity to the Raf proto-oncogene from mouse". This statement is demonstrably false. Unfortunately, the only other paper on ISR1 (Mehlgarten 2007) repeats this claim, adding credibility to it. This manuscript should break this chain of errors and not repeat it anymore. ISR1 is NOT related to Raf. Mammalian Raf kinases are members of the tyrosine kinase-like subfamily (Manning 2002). Yeast does not contain any members of this family. ISR1 is not the orthologue of Raf. Of the 117 kinases in yeast, 79 show greater homology to the kinase domain of mouse Raf than ISR1. The 30% identity reported in the Miyahara paper is extremely misleading since that homology is limited to a 109 residue peptide and not the entire kinase domain. The misinformation about a Raf-ISR1 connection needs to stop now. Further, the introduction should delete the whole paragraph on Raf kinase and Ras signaling. A more accurate and factually correct description of ISR1 and its place in the kinomes of yeast and humans would be greatly appreciated. ISR1 is an unusual kinase and has variant sequences in the place of what are usually invariant kinase signature motifs. For instance, the catalytic aspartate that these authors mutate (D280) is usually in the "HRD" motif. In ISR1, this sequence is HGD. Because of its unusual sequence, many databases list ISR1 as a "putative protein kinase". That is

much more accurate and pertinent to this paper than any discussion of Raf. The connection of ISR1 to Raf and Ras signaling reappears in the discussion where the authors note that the closest relative of ISR1 in S. pombe is the kinase byr2. This is true, but the similarity is very weak and not reciprocal. S. pombe byr2 is more likely to be the orthologue of S. cerevisiae Ste11. If you BLAST pombe byr2 against the S. cerevisiae proteome, the top hit is STE11, not ISR1. In fact, 103 of the 117 yeast kinases show stronger homology to pombe byr2 than does ISR1. S. pombe does not have a clear orthologue of ISR1; there is no connection to Raf or RAS. This paragraph in the discussion should be removed.

Reviewer 1's points are well taken and we agree it might be prudent to remove references to RAF kinase. We have now amended these sections of the manuscript as follows:

a) We have removed the phrase "related to mammalian Raf and" from the abstract
b) We have removed the entire paragraph on Raf kinase and Ras signaling in the introduction.
c) We have added the sentence "Isr1 is annotated as a putative protein kinase due to an unusual catalytic motif, HGD, in place of the signature HRD motif in its kinase domain" to the first paragraph of the results before explaining the kinase dead mutant that we made.
d) We have removed the paragraph discussing Raf from the discussion.

2. Staurosporine. ISR1 will be forever linked to staurosporine since ISR stands for "Inhibition of Staurosporine Resistance". Miyahara et al. in 1998 describe staurosporine as "a potent inhibitor of protein kinase C". This may be true but it is not the whole story. We now know that staurosporine is a potent inhibitor of almost all protein kinases. It binds to the ATP binding pocket with higher affinity than ATP. In fact, its presence in the ATP pocket stabilizes kinase domain structures explaining why this compound is included during crystallization of so many protein kinases. Probably the most relevant data in the Miyahara paper is that isr1 mutants exacerbate a pkc1 mutant, thus connecting both kinases to the cell wall integrity pathway. Perhaps when referring to staurosporine as a PKC inhibitor, they could add that this compound is really a broad spectrum kinase inhibitor.

We agree that it is important to remind the reader that staurosporine inhibits other kinases as well. Therefore, we have changed "whose overexpression sensitized cells to staurosporine, a Pkc1 inhibitor" to "whose overexpression sensitized cells to staurosporine, a broad specificity kinase inhibitor that targets Pkc1."

3. PSTAIR. Many western blot experiments in this paper use PSTAIR antibody as a loading control (Figs 1,5 and 6). This needs to be explained. My understanding is that PSTAIR is a monoclonal that recognizes the PSTAIR sequence in cdk kinases. Yeast has 2 cdk's with the PSTAIR sequence, Cdc28 and Pho85. These two proteins have similar molecular weights: 34.0 vs 34.9 kDa. What is being recognized in the PSTAIR blots? What is the MW range being shown in the PSTAIR panels? This may be an odd control blot to choose since Pho85 becomes an important player in

the control of ISR1 abundance. There is no mention of the PSTAIR panel in the figure legends.

Our laboratory has historically used Cdc28 as a loading control, as it is stable and its levels are not altered significantly in stress or by cell cycle position in growing cells. The antibody we used for this is no longer available, so we have switched to a PSTAIR antibody, which also recognizes Cdc28. The antibody was raised against the sequence (EGVPSTAIREISLLKE) which is 100% identical to the PSTAIR of Cdc28, and 88% identical to the PSTAIR sequence of Pho85. While this antibody is commonly thought of as specific for Cdk1 and used as a loading control, it may well have some activity against Pho85. However, as shown in Figure 6B, deletion of *PHO85* does not obviously change the reactive band. Moreover, like Cdc28, Pho85 is thought to be a stable protein whose levels are not altered during the cell cycle. Thus, we feel that this loading control will easily allow the reader to ascertain that the differences seen for the critical bands are not due to uneven loading of the samples. We have added this explanation of the loading control to figure legends 1,5 and 6: "PSTAIR is a monoclonal antibody that recognizes the PSTAIR sequence in Cdc28 and is shown as a loading control".

4. ISR1 deletion details. The authors note that the isr1Δ:KAN allele in the MATa collection has RNA processing phenotypes caused by interference with the adjacent gene. They make a new isr1Δ strain with a different marker. It would be worth including the details of how their new deletion allele differs from the KAN allele. How did they make an isr1 deletion that doesn't interfere with the adjacent gene?

This is an excellent question. We used a HygMX cassette whereas the deletion collection utilizes a KANMX cassette. Theoretically, both deletions were made in the same way utilizing homology arms that match the promotor and 3'UTR of Isr1 on either side of the deletion cassette. In addition to the different antibiotic marker, our deletion cassette also lacks a tag sequence that the deletion collection included for sequencing purposes. Since the end of the coding region of Isr1 functions as the 3'UTR for Yth1 (this gene is transcribed in the opposite direction), we hypothesize that either this tag sequence in the deletion collection allele is a poor 3'UTR *or* the end of the Hyg sequence is a more stable 3'UTR than the Kan sequence.

We have expanded the text in the results section explaining to the deletion phenotypes as follows:

"The end of the *ISR1* coding sequence overlaps with the 3'UTR of its neighboring gene, *YTH1*, which is an essential component of the mRNA cleavage and polyadenylation factor. Thus, the cordycepin sensitivity of the *isr1*Δ strain from the deletion collection is likely a result of a reduction in function of *YTH1*. We speculate that the HygMX sequence used here might function as a more stable 3'UTR for Yth1 than the KanMX sequence used to generate the *isr1*Δ strain in the deletion collection."

5. Improving the discussion. I have noted above some topics that should be removed from this paper. The one topic that should be added is a better discussion of how

their new data relates to the data in the Mehlgarten paper. That paper is the best description of ISR1 to date and connects ISR1 to the cell wall and PKC1. It's a bit unfair to the Mehlgarten authors to call ISR1 a "previously uncharacterized kinase" (line 418). Poorly characterized, yes; uncharacterized, no.

We appreciate the reveiwer's suggestions and have deleted all mention of Raf/Ras signaling from the discussion. We have replaced "previously uncharacterized kinase" with "poorly characterized kinase" and have added the paragraph below:
"ISR1 was previously implicated in cell wall homeostasis by Mehlgarten et al.'s observation that *ISR1* overexpression results in zymocin resistance, a cell wall stress agent. The findings of that paper suggested a role for Isr1 in chitin biology and are in agreement with the function we have uncovered for *ISR1* as a negative regulator *GFA1*. Mehlgarten et al. delete *PHO85* and find this abolishes the *ISR1* overexpression phenotype, leading the authors to posit that Pho85 activates Isr1. However, our results are directly contrary to that hypothesis and we suggest that the rescue that Melgarten et al. observe is due to the pleiotropic effects that PHO85 has on the cell wall."

Reviewer #2: Review PloS Genetics
Although protein glycosylation is an essential and well-studied cellular process, how protein glycosylation might be regulated throughout the cell cycle and in response to stress is not clear. Here, this report focuses on a protein kinase and negative regulator of an early step in protein glycosylation, which is the biosynthesis of an N-linked sugar precursor. This negative regulatory kinase is regulated by the alternate CDK Pho85, which allows cells to alter the levels of the regulator according to the cell's nutrient needs.

There are many strengths to this study. It is very well written. The data is clear and carefully interpreted. The findings are impactful and presented in a clear and logical manner. The combination of phenotypic and genetic data with immunoblot and MASS SPEC data, plus previous data from the lab and the community lends credence to the study.
The main piece of data that is missing is direct demonstration that Isr1 phosphorylates Gfa1. Typically, this can be shown in two ways, a shift in protein levels by immunoblot (e.g. phos-tag) analysis (which itself is indirect) and by in vitro kinase assays. The authors do present MASS SPEC data to address this point.

Below are some minor comments.
1. Are there homologs to Isr1?
Not according to reviewer 1. While byr2 and mammalian RAF have been shown to have similarity to Isr1, on further analysis, we agree with reviewer 1's assertion that this relationship is tentative and therefore best left out of the manuscript.

2. Immunoblots can be quantitated. By adjusting levels to the loading control, setting it to 1 and comparing other levels to that (Fig. 1B). Numbers can be represented below the IBs.

We have now quantified the Western blots for Figures 1 and 5 and have included these in the new versions of the Figures.

3. What is the significance of the fact that the levels of overexpressed ISR1 are the same as Pkc1 in the cell?
Over-expression with the Gal1 promoter can sometimes generate incredibly high levels of protein. This panel was aimed to convey to readers that, while we have over-expressed this kinase, it is still present at a level consistent with that of other signaling molecules within the cell. We have now quantified this western and clarified this in the text with the statement:

"The expression level of Isr1 from a 2μ plasmid utilizing the *ISR1* promoter (referred to as 2μ *ISR1*) is low compared to GAL-2μ *ISR1,* and even GAL-2μ *ISR1* does not result in extremely high levels of Isr1 (Fig 1B). To confirm this, we empirically determined the levels of Isr1 achieved by the GAL- 2μ *ISR1* construct. We epitope tagged Pkc1 expressed under its endogenous promoter using a 3x-Flag tag, the same epitope tag used for Isr1, and compared these proteins by western blot. This comparison demonstrates that GAL-2μ Isr1 protein levels are similar to the endogenous expression level of Pkc1 (Fig 1C)."

4. Section heading and within the paragraph "Isr1 overexpression is synthetic lethal with enzymes in the hexosamine biosynthesis pathway' should be synthetically
We thank the reviewer for pointing this out. We have now corrected this typo.

5. Does the fact that Gfa1-3A cells are sensitive to tunicamycin imply that phosphorylation of Gfa1 is important for balancing the cell's protein glycosylation needs?
We feel like the most likely explanation for this sensitivity is that the *GFA1-3A* allele is hypomorphic for normal activity. Consistent with this, *GFA1-3A's* tunicamycin sensitivity is recessive to wild type. Modest loss of function is a commonly seen for phosphosite mutants (and epitope tags) when assays are used that require full activity of the mutated protein. Importantly, *GFA1-3A's* resistance to *ISR1* over-expression is dominant, suggesting that this resistance is due to a gain of function. We have now added an additional sentence in the discussion to make this clearer.

6. Given that Isr1 is cell cycle regulated, it might be phosphorylated by the cell's major CDK, Cdc28?
We agree that the phosphodegron is likely to be additionally targeted Cdc28. Experiments not included here suggest that Cdc28 may target Isr1 during a mitotic arrest. However, we were unable to observe this under physiologic conditions, so these experiments were not included.

7. Plate labels should appear above not below the pictures.
We appreciate this suggestion. A brief survey of the literature suggests that both conventions are used. We have opted for below the plates. However, if the editors have a strong preference, we will gladly re-adjust these figures.

8. In Figure 5, it looks like cdc4-1 is much more important than cdc53-1 in stabilization of Isr1 levels. Again, quantitation of protein levels might help this analysis.
We have now quantified the data in Figure 5, and have included these data in the new version of Figure 5. The quantification shows that the stabilization is actually quite similar in both of these strains. However, it is important to note that experiments comparing alleles of essential genes will yield different results simply due to the fact that the alleles are of different strengths. Thus, it is impossible to judge the relative contributions of Cdc4 and Cdc53 from an experiment such as that presented in Figure 5 due to the fact that no single allele is entirely disabled at the none-restrictive temperature. Instead, we can only show that each mutant is stabilized compared to wild-type.

9. wildtype should be wild-type
We thank the reviewer for this suggestion. We have corrected this throughout.

10. What do the authors think about all the other proteins that are being phosphorylated by Isr1?
From the experiment in Figure 4D, we feel that Gfa1 may be the most important substrate of Isr1. While other substrates were not obvious from the mass spectrometry, other substrates could contribute to the ISR1 mutation or overexpression phenotypes. We tested other kinases that were phosphorylated in the mass spectrometry dataset to determine if they might be relevant for the phenotype (now included in the supplement), but deleting these kinases had no effect on the Isr1 phenotype.

11. Are the glycosylation profiles of select glycosylated proteins impacted by these mutants, observed for example by IB analysis?
This is an interesting line of investigation. We did not observe any obvious changes in mobility of glycosylated substrates.

Reviewer #3: This is a well-written and compelling story that reveals a function for the yeast Raf homolog, Isr1. Deletion of ISR1 confers tolerance to N-glycosylation inhibitor tunicamycin, whereas its moderate overexpression confers tolerance to the chitin antagonist calcofluor white. Based on these phenotypes, the authors pursued the possibility that ISR1 is a negative regulator of hexosamine biosynthesis, specifically UDP-N-acetyl glucosamine (UDP-GlcNAc) production by GFA1. UDP-GlcNAc is required for the first steps in N-glycosylation and GPI-anchor biosynthesis, as well as for the production of chitin. The authors showed that strong ISR1 overexpression causes growth inhibition, which was suppressed either by over-expression of GFA1, or by addition of glucosamine to the medium, which is scavenged to produce UDP-GlcNAc independently of GFA1.

The authors go on to show that Gfa1 is phosphorylated on neighboring residues in an Isr1-dependent manner, mutation of which to Ala (GFA1-3A) overcame the growth inhibition associated with overexpression of ISR1, supporting the conclusion that Isr1 phosphorylation of Gfa1 is inhibitory. One result that was not entirely

consistent with this conclusion was the finding that the GFA1-3A mutation did not confer tolerance to tunicamycin (Fig. 4E), as would be expected if it was to phenocopy deletion of ISR1. The authors explain this with a demonstration that the GFA1-3A mutation is somewhat hypomorphic and causes tunicamycin sensitivity by itself. They further suggest that Isr1 has other targets that contribute to its role in N-glycosylation. This is a reasonable conjecture.

Finally, the authors show that the N-terminal regulatory domain of Isr1 contains a phosphodegron that is phosphorylated by Pho85 and targeted by the SCF-Cdc4 ubiquitylation complex. This confers tight cell cycle regulation of Isr1 levels so that its activity is largely confined to the G1/S transition. These experiments set out in Fig. 5, should be accompanied by quantitation of the immunoblot bands. Aside from this lone criticism, this manuscript is viewed to be very strong.

We thank the reviewer for their insightful review. We have now quantified Figure 5 and included this in the new version of the manuscript in Figure 5.
